# Telehealth Modality Preferences for Video and Voice-Only Visits Among US Clinicians and the Public: Cross-Sectional, Web-Based Survey Study

**DOI:** 10.2196/72276

**Published:** 2025-06-13

**Authors:** Rachel C Forcino, Julie K Johnson, Glyn Elwyn

**Affiliations:** 1 Department of Population Health, University of Kansas School of Medicine, 3901 Rainbow Blvd, Mail Stop 1008, Kansas City, KS, 66160, United States, 1 913-588-4742; 2 Department of Surgery, University of North Carolina at Chapel Hill, Chapel Hill, United States; 3 The Dartmouth Institute for Health Policy and Clinical Practice, Geisel School of Medicine, Dartmouth College, Hanover, United States

**Keywords:** telehealth, acceptability, survey research, patient-centered care, preferences

## Abstract

**Background:**

The onset of the COVID-19 pandemic led US health systems to rapidly implement telehealth services to connect patients and clinicians. This rapid expansion of telehealth allowed us to explore how a telehealth experience may be best delivered across populations and contexts.

**Objective:**

We aimed to identify telehealth modality preferences (voice vs video) among clinicians and the populations they serve and explore the barriers to telehealth use from clinician and public perspectives.

**Methods:**

We conducted 2 independent, cross-sectional, web-based surveys. US physicians specializing in internal medicine, psychiatry, urology, orthopedic surgery, or obstetrics and gynecology completed a 23-item survey through Sermo’s research panel. Quotas ensured equal representation across the selected clinical specialties. Adult members of the US general public completed a 26-item survey through Qualtrics’ research panel. Quotas ensured the general public sample approximated the US population on educational attainment, gender, and rural residence.

**Results:**

We recruited 253 clinician participants and 418 general public participants in September 2020, with survey completion rates of 22% (253/1139) and 93% (418/451), respectively. For the initial encounter, 85% (216/253; 95% CI 80%‐89%) of clinicians and 51% (215/418; 95% CI 47%‐56%) of public participants preferred video over voice only. In multiple logistic regression analyses, members of the public with broadband internet at home were more likely than those without broadband to prefer video over voice only for a first visit with a new clinician (57% vs 40%; odds ratio 2.09, 95% CI 1.25‐3.49). In an established clinical relationship, 63% (160/253; 95% CI 57%‐69%) of clinicians and 33% (137/418; 95% CI 28%‐38%) of the general public preferred video over voice only when discussing a new clinical problem. For a follow-up visit, only 26% (65/253; 95% CI 20%‐32%) of clinicians and 28% (117/418; 95% CI 24%‐33%) of the general public preferred video over voice only. Clinicians and the general public agree that technology not working properly is their main source of telehealth frustration (86/249, 35% of clinicians; 62/220, 28% of public with telehealth experience). Other major frustrations include limitations on what content can be included in the visit (main frustration for 83/249, 33% of clinicians; 54/220, 25% of the public) and downloading new technology (52/220, 24% of the public).

**Conclusions:**

Although telehealth connections with video are increasingly common, important factors are associated with a preference for voice-only telehealth connections. Clinicians prefer video over voice-only connections more than patients do for new clinical relationships and new clinical problems. For follow-up care, both clinicians and the public prefer voice-only telephone visits over video. Barriers to video-enabled telehealth experiences include technology failures, varied technology platforms across providers, and a need for more reliable high-speed internet connection. Voice-only telephone-mediated services can potentially improve health care access and experiences in light of these barriers to video-based care.

## Introduction

The worldwide spread of COVID-19 pandemic led to a vast increase in the use of telehealth to connect patients and clinicians while limiting potential viral exposure [1]. While long-standing research initiatives have explored key facets of telehealth in some contexts [2-7], telehealth’s previously limited scope and numerous barriers to widespread use have limited detailed consideration of how to routinely deliver an equitable and accessible telehealth experience across diverse populations and contexts. More specifically, satisfaction with video-enabled telehealth is generally high [8], but there has been limited investigation into telehealth modalities and how patient and clinician characteristics may shape preferences for video-enabled versus voice-only telehealth experiences.

Most previous research comparing telehealth modalities has been conducted in a single clinical context and has compared patient and clinician satisfaction between video-enabled and audio-only telehealth modalities [9-13]. Less is known about drivers of patients’ and clinicians’ telehealth modality preferences, especially across clinical contexts. Theoretical frameworks, such as diffusion of innovations and the theory of planned behavior, link the uptake of new technologies at both individual and organizational levels to preferences, that is, attitudes and subjective norms surrounding those technologies [14,15]. The current gap in knowledge surrounding drivers of telehealth modality preferences represents a critical gap in understanding potential constraints on telehealth access, designing equitable telehealth services, and establishing adequate supports for those services.

During the COVID-19 pandemic, which initiated unprecedented use of telehealth services in the United States, we aimed to identify telehealth modality preferences (voice-only telephone vs video) among clinicians and the populations they serve and explore barriers to telehealth use from clinician and public perspectives.

## Methods

### Design

#### Overview

We designed and reported this survey study according to the Checklist for Reporting Results of Internet E-Surveys (CHERRIES) [16]. We administered independent, cross-sectional, web-based surveys to the following internet survey panel members: (1) clinicians practicing in the United States and (2) adult members of the public living in the United States. Survey items were developed through a literature review and research team consensus. Research team members then reviewed and pretested the electronic surveys with colleagues to assess functionality and readability. We did not formally assess the survey instrument’s validity or reliability. Refer to MultimediaAppendices 1  2 for the full questionnaires.

#### Physician Survey

The physician survey included 23 multiple-choice items. Out of them, 6 items assessed demographic characteristics, including clinical specialty, rural or urban setting, use of an electronic health record, age, and years since medical school graduation. A total of 3 items asked respondents their preferred telehealth modality for (1) attending a first visit with a patient they do not know; (2) hearing from a patient they know about a new problem; and (3) hearing from a patient they know about a problem they have talked about before. In addition, 7 subsequent items asked respondents to rate the importance of selected telehealth features. In total, 6 subsequent items asked respondents to rate frustration with 5 aspects of telehealth, plus an “other” option. Within each of these batteries, we randomized item order to reduce the risk of order effects. An open-ended response field was available for any other comments about telehealth.

#### Public Survey

The public survey included 26 multiple-choice items and one open-ended item. In total, 10 items assessed demographic characteristics, including educational attainment, rural or urban setting, gender, health literacy, age, employment status, telecommunications services at home, internet use frequency, and experience with telehealth in the past year. Using the same items as in the physician survey, 3 items asked respondents their preferred telehealth modality for attending a first visit with a clinician they do not know, telling a clinician they don’t know about a new problem, and updating a clinician they know about a problem they’ve talked about before. A total of 7 items asked respondents to rate the importance of selected telehealth features, and 6 items explored frustrations with telehealth. We randomized item order within each battery. An open-ended response field was available for any other comments about telehealth.

### Participants

#### Clinician Survey

Participants included physicians practicing in the United States in one of the following specialties: (1) internal medicine; (2) psychiatry; (3) urology; (4) orthopedic surgery; or (5) obstetrics and gynecology. In selecting these specific medical and surgical specialties, we sought to capture variation in the clinical activities required to conduct routine patient care, including physical examination, imaging, laboratory testing, and detailed bidirectional discussion. We recruited participating clinicians through Sermo’s research panel and used quotas to ensure equal representation across the selected clinical specialties. Following an established rule of thumb recommending 50 participants per key subgroup, we sought to recruit 50 clinicians in each clinical specialty [17].

#### Public Survey

Participants included adult (aged 18 years and older) US residents recruited through Qualtrics’ research panel. We used recruitment quotas to ensure the sample approximated the US population on educational attainment, gender, and rural residence. We ensured representation across education levels as a component of socioeconomic status (SES), as SES is associated with uptake of video-enabled telehealth services [18]. We ensured representation of rural residents because some telecommunications services, such as high-speed internet and cell phone service coverage, are not widely available or consistently reliable in some rural areas of the United States.

### Procedure

Each survey began with a research study information sheet describing the purpose of the study, voluntary nature of participation, survey duration, data storage and privacy considerations, and contact information for the principal investigator. Responses were required for all multiple-choice survey items, and respondents could not review and change their answers. Upon completion of the survey, physician respondents received a US $13 honorarium directly from the Sermo research panel. Due to the sample vendor’s recruitment strategy, public respondents received gift card honoraria of varying amounts, which were agreed with the participant before survey initiation and delivered directly by Qualtrics’ research panel.

### Analysis

Our quota-based sampling strategy promoted variation in clinical specialty and rural or urban location among clinicians and ensured our public sample approximated the US population; we therefore did not weight the survey data. We excluded physician survey responses that were less than 60 seconds in duration and public responses that were less than 75 seconds in duration, considering the minimum time required to read and complete survey items.

To identify telehealth modality preferences among clinicians and the public, we conducted 6 multiple logistic regression analyses with the independent variables of a preference for video among clinicians and the public, respectively, in each of the following scenarios: (1) first visit with a new patient or clinician; (2) discussing a new problem with a known patient or clinician; and (3) discussing an existing problem with a known patient or clinician. Physician analyses are controlled for rural or urban locations, clinical specialty, age, years since medical school graduation, and work settings. Explanatory variables in the public analyses included rural or urban location, age, employment status, less than daily internet access, health literacy, access to 5 types of telecommunications services at home, and previous experience with video- and phone-based telehealth. We selected the largest anticipated level as the reference group a priori for each nonordinal categorical explanatory variable in the logistic regression analyses. We conducted all statistical analyses using Stata (version 13; StataCorp) software.

To explore barriers to telehealth use from physician and public perspectives, we calculated frequencies and descriptive statistics, including binomial exact confidence intervals, within each sample to identify telehealth frustrations and key telehealth features. For the single open-ended item collecting further comments on telehealth, 2 members of the research team (RCF and JKJ) independently and inductively coded approximately 25% of the responses, met to reconcile any differences in coding and to identify patterns, then independently completed coding before collaboratively agreeing on major themes [19].

### Ethical Considerations

This research was conducted in accordance with the Declaration of Helsinki. The Dartmouth College Committee for the Protection of Human Subjects reviewed this study and designated it exempt from further review (no. #32127). All survey participants reviewed a study information sheet approved by our institutional research ethics committee before their participation.

## Results

### Participants

#### Clinician Survey

In September 2020, a total of 1186 clinicians accessed the first page of the survey, 1139 clinicians completed the research consent stage, and 260 proceeded to complete all survey items. We included 253 responses in analyses following data quality checks, representing a completion rate of 22.2% (253/1139). Participants included 51 internal medicine physicians, 50 psychiatrists, 50 urologists, 52 orthopedic surgeons, and 50 obstetrician gynecologists. Most clinician participants were aged between 35 and 64 years (205/253, 81%), completed medical school between 10 and 29 years ago (158/253, 62%), and lived in a large city (108/253, 43%) or a suburb near a large city (99/253, 39%).

#### Public Survey

In September 2020, a total of 459 members of the public accessed the first page of the survey, 451 completed the research consent stage of the survey, and 439 completed all survey items. Following data quality checks, we included 418 responses in analyses, representing a completion rate of 92.7% (418/451). A total of 63% of participants (262/418) had broadband internet access at home. While 83% (346/418) had cell phone service, only 52% (216/418) subscribed to mobile data and internet service.

Table 1 presents demographic profiles of physicians and public participants.

**Table 1.** Participant characteristics.

Characteristics	General public (n=418), n (%)	Clinicians (n=253), n (%)
Age (years)		
18‐24	6 (27)	—^a^
25‐34	18 (77)	9 (23)
35‐44	21 (88)	31 (79)
45‐54	19 (78)	26 (66)
55‐64	15 (61)	24 (60)
65+	20 (87)	10 (25)
Residence (public) or workplace (clinicians)		
Large city	28 (117)	43 (108)
Suburb near a large city	34 (142)	39 (99)
Small city or town	18 (76)	15 (39)
Rural area	20 (83)	3 (7)
Gender		
Men	50 (209)	—
Women	49 (204)	—
Nonbinary or prefer not to say	1 (5)	—
Employment status		
Full time paid work	50 (208)	—
Part time paid work	12 (50)	—
Unemployed	10 (42)	—
Retired	22 (94)	—
Other	6 (24)	—
Work setting		
Private or solo practice	—	20 (50)
Group practice	—	47 (119)
Hospital- or health maintenance organization–based practice	—	31 (79)
Other	—	2 (5)
Frequency of internet use		
Several times a day	82 (344)	—
About once a day	7 (29)	—
Less often than once a day	11 (45)	—
Educational attainment		
Up to high school diploma or equivalent	22 (92)	—
Technical training	5 (22)	—
Some college	21 (86)	—
College degree	34 (143)	—
Graduate degree	18 (75)	100 (253)
Services at home		
Broadband internet	63 (262)	—
Dial-up internet	7 (31)	—
Mobile data	52 (216)	—
Cell phone	83 (346)	—
Landline phone	33 (136)	—
Clinical specialty		
Internal medicine	—	20 (51)
Psychiatry	—	20 (50)
Urology	—	20 (50)
Orthopedic surgery	—	21 (52)
Obstetrics and gynecology	—	20 (50)
Years since medical school graduation (years)		
0-9	—	16 (41)
10-19	—	35 (89)
20-29	—	27 (69)
30 or more	—	21 (54)

a Not applicable.

### Modality Preferences

#### Clinicians

For a first meeting with a new patient, 85% (216/253; 95% CI 80%‐89%) of clinicians preferred video over voice-only telephone (Table 2), with no significant differences in modality preferences observed between subgroups (refer to Table 3 for full adjusted logistic regression results). To discuss a new problem with a known patient, 63% (160/253; 95% CI 57%‐69%) of clinicians preferred video over voice-only telephone. To follow up with a known patient about a previously discussed problem, only 26% (65/253; 95% CI 20%‐32%) of clinicians preferred video.

**Table 2.** Responses for the question “Which platform would you prefer for each of the following scenarios?”

Items and responses			Respondents, n (%)
Clinicians			
	Attending first appointment with a patient whom I don’t know		
		Phone	18 (7)
		Video	216 (85)
	Hearing from a patient whom I know about a new problem		
		Phone	52 (21)
		Video	160 (63)
	Hearing from a patient I already know about a problem we’ve talked about before		
		Phone	137 (54)
		Video	65 (26)
Public			
	Attending first appointment with a clinician whom I don’t know		
		Phone	203 (49)
		Video	215 (51)
	Telling a clinician whom I know about a new problem		
		Phone	281 (67)
		Video	137 (33)
	Telling a clinician whom I know about a problem we’ve talked about before		
		Phone	301 (72)
		Video	117 (28)

**Table 3.** Multiple logistic regression results: clinician characteristics and preference for video visits.

	First visit with new patient, odds ratio (95% CI)	New problem with known patient, odds ratio (95% CI)	Existing problem with known patient, odds ratio (95% CI)
Clinical specialty			
Internal medicine	Reference group	Reference group	Reference group
Psychiatry	1.29 (0.35‐4.73)	0.67 (0.26‐1.72)	1.47 (0.57‐3.78)
Urology	1.43 (0.40‐5.13)	0.56 (0.22‐1.42)	1.40 (0.54‐3.62)
Orthopedic surgery	0.64 (0.21‐1.93)	0.57 (0.23‐1.43)	0.87 (0.33‐2.30)
Obstetrics and gynecology	0.66 (0.22‐2.02)	0.16 (0.07‐0.41)^a^	1.13 (0.43‐2.92)
Years since medical school graduation (years)			
0-9	2.24 (0.16‐30.92)	0.59 (0.10‐3.57)	1.82 (0.28‐11.77)
10-19	0.50 (0.07‐3.36)	0.28 (0.07‐1.15)	3.64 (0.80‐16.60)
20-29	0.38 (0.09‐1.64)	0.42 (0.15‐1.17)	1.27 (0.41‐3.95)
30 or more	Reference group	Reference group	Reference group
Work setting			
Private or solo practice	2.39 (0.75‐7.67)	2.17 (0.92‐5.12)	1.38 (0.57‐3.35)
Group practice	2.10 (0.91‐4.84)	2.05 (1.05‐3.99)^a^	1.34 (0.65‐2.77)
Hospital- or health maintenance organization–based practice	Reference group	Reference group	Reference group
Age (years)			
25‐34	0.19 (0.01‐4.98)	3.33 (0.42‐26.46)	—^b^
35‐44	0.49 (0.03‐7.45)	9.08 (1.66‐49.70)^a^	0.96 (0.15‐6.08)
45‐54	0.33 (0.03‐3.76)	3.98 (0.97‐16.26)	1.14 (0.22‐5.84)
55‐64	0.34 (0.04‐3.14)	1.90 (0.64‐5.60)	1.38 (0.37‐5.11)
65+	Reference group	Reference group	Reference group
Work location			
Large city	Reference group	Reference group	Reference group
Suburb near a large city	0.88 (0.39‐2.03)	1.14 (0.59‐2.20)	1.15 (0.58‐2.29)
Small city, town, or rural area	1.86 (0.55‐6.32)	1.07 (0.48‐2.38)	0.78 (0.32‐1.94)

a Statistically significant α=.05.

b Not applicable.

#### Public

When first meeting with a new clinician, public participants were evenly split between a preference for video (51%, 215/418; 95% CI 47%‐56%) versus voice-only telephone. Those with broadband internet (odds ratio [OR] 2.09, 95% CI 1.25‐3.49) at home were twice as likely as others to prefer video for this first visit. When seeing a familiar clinician to discuss a new clinical problem, 33% (137/418; 95% CI 28%‐38%) of public participants preferred video over voice-only telephone. Participants with mobile data access at home were more likely than their peers without mobile data (OR 2.37, 95% CI 1.44‐3.91) to prefer video to discuss new clinical problems with familiar clinicians. For a follow-up visit with a familiar clinician about a previously-discussed problem, 28% (117/418; 95% CI 24%‐33%) of public participants preferred video, with 72% (301/418; 95% CI 67%‐76%) preferring voice-only telephone. Participants with limited health literacy as measured by the single-item literacy screener [20] (OR 2.2, 95% CI 1.24‐3.91) and those who access the internet on less than a daily basis (OR 2.83, 95% CI 1.15‐6.96) were more likely than others to prefer video for follow-up visits. Across all 3 visit types, people with previous video-enabled telehealth experience were more likely than their peers to prefer video over phone-based telehealth visits (Table 4).

**Table 4.** Multiple logistic regression results: public characteristics and preference for video visits.

Characteristics	First visit with new clinician, odds ratio (95% CI)	New problem with known clinician, odds ratio (95% CI)	Existing problem with known clinician, odds ratio (95% CI)
Residence			
Large city	Reference group	Reference group	Reference group
Suburb near large city	0.95 (0.52‐1.75)	1.60 (0.85‐3.01)	0.92 (0.47‐1.77)
Small city or town	1.26 (0.61‐2.60)	1.22 (0.56‐2.65)	0.93 (0.42‐2.06)
Rural area	0.65 (0.30‐1.40)	1.25 (0.55‐2.84)	0.78 (0.33‐1.84)
Paid employment			
Working	Reference group	Reference group	Reference group
Not currently working	0.97 (0.54‐1.75)	1.08 (0.59‐2.00)	0.84 (0.44‐1.59)
Sex			
Male	Reference group	Reference group	Reference group
Female	1.42 (0.82‐2.46)	1.06 (0.59‐1.91)	0.91 (0.50‐1.68)
Age (years)			
18‐24	0.43 (0.12‐1.52)	2.30 (0.63‐8.32)	3.55 (0.98‐12.83)
25‐34	0.56 (0.21‐1.46)	0.80 (0.29‐2.15)	0.36 (0.12‐1.05)
35‐44	1.08 (0.42‐2.75)	1.83 (0.70‐4.78)	1.15 (0.42‐3.12)
45‐54	0.47 (0.21‐1.05)	0.97 (0.42‐2.28)	0.57 (0.22‐1.46)
55‐64	0.71 (0.32‐1.57)	0.77 (0.33‐1.79)	0.74 (0.30‐1.81)
65+	Reference group	Reference group	Reference group
Less than daily internet use	1.31 (0.55‐3.12)	1.80 (0.74‐4.38)	2.83 (1.15-6.96)^a^
Previous telehealth visit by phone	0.77 (0.48‐1.26)	0.75 (0.44‐1.26)	0.65 (0.37‐1.15)
Previous telehealth visit by video	3.75 (2.28-6.15)^a^	3.57 (2.18-5.84)^a^	2.92 (1.73-4.90)^a^
Lower health literacy (Morris et al [20])	0.71 (0.42‐1.18)	0.99 (0.58‐1.70)	2.20 (1.24-3.91)^a^
Landline phone at home	0.77 (0.47‐1.27)	1.11 (0.65‐1.87)	1.00 (0.57‐1.77)
Cell phone at home	1.91 (0.98‐3.72)	1.81 (0.87‐3.77)	1.02 (0.49‐2.14)
Mobile data at home	1.07 (0.68‐1.69)	2.37 (1.44-3.91)^a^	1.36 (0.81‐2.29)
Dial-up internet at home	1.04 (0.43‐2.51)	0.44 (0.16‐1.26)	0.30 (0.09-1.00)^a^
Broadband internet at home	2.09 (1.25-3.49)^a^	1.17 (0.67‐2.04)	1.20 (0.66‐2.20)

a Statistically significant α=.05.

### Telehealth Frustrations

#### Clinicians

Clinicians’ primary frustrations with telehealth were technology not working properly (single main frustration for 86/249, 35% of respondents), limitations on what can be covered in the visit (83/249, 33%), and helping patients understand how to use the technology (56/249, 22%; Table 5).

**Table 5.** Responses for the question “What has been your main frustration with telehealth over the past few months?”

Responses	Public, n (%)	Clinicians, n (%)
Technology not working properly	62 (28)	86 (35)
Limitations on what we can cover in the visit	54 (25)	83 (33)
Downloading new technology	52 (24)	5 (2)
Figuring out how to use new technology	44 (20)	16 (6)
Helping patients understand how to use the technology	0 (0)	56 (22)

#### Public

Among the general public, technology not working properly was the top frustration with telehealth (62/220, 28% with telehealth experience) followed by limitations on what can be covered in the visit (54/220, 25%), downloading new technology (52/220, 24%), and figuring out how to use new technology (44/220, 20%).

### Priority Telehealth Features

Being able to hear and be heard clearly by the other party was the most important telehealth feature for both clinicians and the general public. A total of 77% (194/253) of clinicians rated being heard clearly as “extremely important” and 76% (193/253) rated hearing clearly as “extremely important.” In total, 64% (266/418) of the general public rated both being heard clearly and hearing clearly as “extremely important.” Ease of using the platform (eg, starting and ending the appointment and controlling the volume) was the second priority for both clinicians (145/253, 57%) and the public (184/418, 44%). Table 6 presents feature ratings for the physician and public samples.

**Table 6.** Responses for the question “How important are each of the following telehealth features to you?”

Items	Clinicians reporting “extremely important,” n (%)	Public reporting “extremely important,” n (%)
The other person being able to hear me clearly	194 (77)	266 (64)
Being able to hear the other person clearly	193 (76)	266 (64)
Ease of using the platform (eg, starting and ending the appointment and controlling the volume)	145 (57)	184 (44)
Being able to see the other person clearly	108 (43)	155 (37)
Flexibility in where I can take the appointment	89 (35)	126 (30)
The other person being able to see me clearly	85 (34)	161 (39)
Being able to [see the clinician’s screen/share my screen] and review materials together	54 (21)	141 (34)

### General Reactions to Telehealth

**Figure 1.**
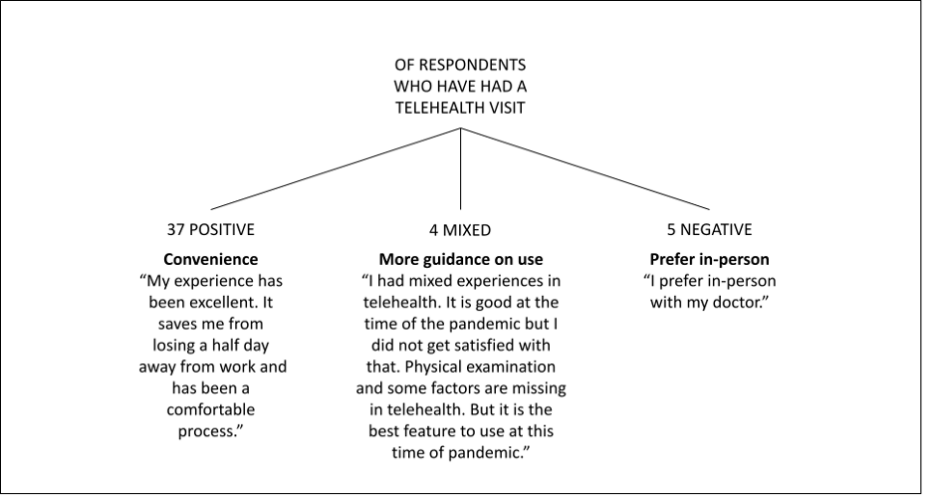
Public sample: is there anything else you’d like to tell us about telehealth?

We identified 3 themes in the written comments: (1) people find telehealth convenient; (2) people need more guidance in using telehealth; and (3) some people still prefer in-person care (Figure 1). Members of the public with previous telehealth experience generally reacted positively to telehealth services during the COVID-19 pandemic, describing its convenience compared with in-person visits. Responses in favor of telehealth cited the lack of required travel and limited time off work as benefits. Few responses indicated mixed or negative reactions to telehealth, citing the lack of a physical exam and a desire for in-person interaction with a clinician.

## Discussion

### Principal Findings

Although clinicians have viewed video-enabled telehealth visits as an important tool during the COVID-19 pandemic, especially for establishing care with new patients, the general public is less enthusiastic about using video for telehealth. Most clinicians favor video for telehealth visits with new patients, but only half of public participants share this preference. For follow-up care, both clinicians and the public prefer voice-only telephone visits over video. Therefore, access to high-quality audio connections must be the top priority in telehealth implementation; equitable implementation of video connections may be a useful secondary step.

Across all included visit types, members of the public with previous experience of a video-based telehealth visit were more likely than their peers to prefer video visits over phone visits, providing a basis for intervention development to help patients become more comfortable with video visits. Previous research has also demonstrated clinicians to be more comfortable using telehealth modalities with which they have previous experience [21]. Members of the public with broadband internet access are also more likely than others to prefer video visits to initiate care with a new clinician, suggesting that inequities in broadband internet availability may exacerbate disparities in telehealth care access. Members of the public with limited health literacy are more likely than their peers to prefer video for follow-up care. Interventions to address disparities could promote both health literacy and technological literacy to improve patients’ telehealth access and experiences.

Primary barriers to telehealth are technological glitches, limitations on what can be covered in a visit, and the burden of downloading and learning new technologies. Despite clinicians’ clear preference for video visits with new patients, their top priorities for telehealth are hearing and being heard clearly. Members of the public share these top priorities.

### Context in Existing Literature

Our findings are consistent with previous literature demonstrating both the general acceptability of telehealth services [8] and a patient preference for telehealth use with a familiar clinician over an unknown clinician [3]. While many members of the public still prefer in-person visits to telehealth [22], we demonstrate that telehealth offers an acceptable and accessible service for those who prefer it. This may include people who face high indirect costs of in-person care, including travel and related time away from work [23]. Patients and clinicians both recognize the advantages of telehealth for bridging logistical barriers to health care, especially when physical co-location is not required. Examples include follow-up conversations and the review of data collected using technology and sensors. The reduced travel costs apply to all patient populations, but are especially relevant for rural-residing patients in particular [24,25].

An important contribution of this study is identifying a stronger preference for video-based telehealth visits among people with limited health literacy in follow-up care, where most others prefer a voice-only telephone call. The visual cues facilitated by video connection may enhance understanding among this often underserved patient population [26,27]. By supporting patients with limited health literacy to access and use video-enabled telehealth services, clinical teams may leverage the benefit of pictorial superiority in this population, since the use of visual cues to communicate health information has been shown to improve patient outcomes, especially among those with lower health literacy [26].

In contrast to our findings, other research in outpatient settings demonstrates that patients accessing video-enabled telehealth are more likely to prefer telehealth for long-term health issues than those accessing voice-only telehealth. This suggests a preference for video over voice-only telehealth for chronic issues and follow-up care. More research is needed into how telehealth preferences are influenced by context and setting and into the impacts of telehealth modality on service delivery and health outcomes.

### Strengths and Limitations

A strength of this study is its exploration of telehealth modality preferences among clinicians and members of the public at a time when telehealth visits have grown exponentially and new telehealth technologies are rapidly proliferating. Including multiple clinical specialties contributes to its representativeness of a broad range of US clinicians. While our sampling strategy leaves us unable to calculate a response rate, the public survey achieved a very high completion rate; 92.9% (418/451) of those who opened the first page of the survey completed it. This high completion rate may have benefited from the short survey duration and the timely, relevant nature of the topic, as it was fielded during the COVID-19 pandemic in September 2020.

Our study is limited by its internet-based panel recruitment strategy. Despite recruitment quotas ensuring minimum subgroup sample sizes, the panel-based samples may preclude true representativeness of the US population of clinicians and members of the public. In particular, completion of the internet-based survey requires some access to and comfort with internet-mediated content. However, to still find preferences for telephone over internet-based video calls in such a sample lends support to those findings. We also collected only limited demographic information on participants in order to limit survey duration and maximize the completion rate; we therefore lack information on some demographic characteristics that may be associated with telehealth modality preferences, including primary language, digital literacy, and relationship or household status [28-30]. Furthermore, we did not conduct psychometric testing of the survey instrument, which may limit the validity of our findings. Although we used plain language in descriptions and item wording and we pretested the questionnaire for face validity, more research is needed to confirm additional aspects of validity and reliability for included survey measures. While responses were required for all multiple-choice survey items, we also established a minimum completion time for all included survey responses, reducing threats to validity associated with participants speeding through required survey items.

### Conclusion

Telehealth brings significant benefits but also has varied mode preferences and raises equity concerns. Despite the potential benefits of video visits over voice-only visits, particularly for people with lower health literacy, lack of access to broadband internet service at home and lack of coordination between providers regarding the adoption of common telehealth technology remain key barriers to widespread public preference for video-based telehealth services.

Future work in this area is required at multiple levels, especially the development and implementation of clinician- and patient-facing educational interventions to support effective telehealth access and delivery, synchronization of telehealth platforms across provider groups to facilitate access, and reimbursement of telehealth services in a way that incentivizes clinical teams and supports telehealth sustainability.

## Supplementary material

10.2196/72276 Multimedia Appendix 1 Clinician questionnaire.

10.2196/72276 Multimedia Appendix 2 Public questionnaire.

## Abbreviations:

CHERRIES: Checklist for Reporting Results of Internet E-Surveys
OR: odds ratio
SES: socioeconomic status
