# Supplementary material for: Telehealth Modality Preferences for Video and Voice-Only Visits Among US Clinicians and the Public: Cross-Sectional, Web-Based Survey Study
Source: J Med Internet Res. 2025 Jun 13;27:e72276. doi: 10.2196/72276 (PMC12180677; doi:10.2196/72276)
Supplement: Multimedia Appendix 1 [file jmir-v27-e72276-s001.docx]

**Appendix 1: Clinician questionnaire**

S1. What is your clinical specialty?

1 Internal medicine [quota=50]

2 Psychiatry [quota=50]

3 Urology [quota=50]

4 Orthopaedic surgery [quota=50]

5 Obstetrics/Gynecology [quota=50]

6 Other (please specify) [TERMINATE]

D5. How would you describe the community where you primarily work?

1 A large city

2 A suburb near a large city

3 A small city or town

4 A rural area [quota=20% of responses]

The COVID-19 pandemic has led to an increase in telehealth, where people have appointments with clinical teams using technology such as telephone or computer. We’d like to ask you a few questions about the way telehealth should be developed in the future.

When we talk about ‘phone calls,’ we mean real-time sound-only connections on a landline or cell phone.

When we talk about ‘videoconferences,’ we mean real-time sound and visual connections on a computer, tablet, or smartphone.

| **Q1. Imagine a world where your reimbursements were the same regardless of whether the clinical encounter is in-person, on the phone, or in a video conference. Which platform would you prefer for each of the following scenarios:** [randomize item order] | | | |
| --- | --- | --- | --- |
|  | **Phone call** | **Videoconference** | **No preference** |
| 1. Attending my first appointment with a patient who I don’t know | 1 | 2 | 3 |
| 1. Hearing from a patient who I know about a new problem | 1 | 2 | 3 |
| 1. Hearing from a patient who I already know about a problem that we’ve talked about before | 1 | 2 | 3 |

| **Q2. How important are each of the following telehealth features to you?** [randomize item order] | | | | | |
| --- | --- | --- | --- | --- | --- |
|  |  | **Not important** | **Somewhat important** | **Important** | **Extremely important** |
| **Q2a.** | Ease of using the platform (e.g. starting and ending the appointment, controlling the volume) | 0 | 1 | 2 | 3 |
| **Q2b.** | Flexibility in where I can take the appointment (e.g. in the office, at home, somewhere else) | 0 | 1 | 2 | 3 |
| **Q2c.** | Being able to hear the patient clearly | 0 | 1 | 2 | 3 |
| **Q2d.** | Being able to see the patient clearly | 0 | 1 | 2 | 3 |
| **Q2e.** | The patient being able to hear me clearly | 0 | 1 | 2 | 3 |
| **Q2f.** | The patient being able to see me clearly | 0 | 1 | 2 | 3 |
| **Q2g.** | Being able to share what’s on my computer screen and review materials together | 0 | 1 | 2 | 3 |

**Q3. Have you had any of the following frustrations with telehealth over the last few months?** (randomize response option order a-d)

|  |  | No frustration | A little frustration | Some frustration | A lot of frustration |  | Not applicable |
| --- | --- | --- | --- | --- | --- | --- | --- |
| Q3a. | Downloading new technology | 0 | 1 | 2 | 3 |  | 99 |
| Q3b. | Figuring out how to use new technology | 0 | 1 | 2 | 3 |  | 99 |
| Q3f. | Helping patients understand how to use the technology | 0 | 1 | 2 | 3 |  | 99 |
| Q3c. | The technology not working properly, e.g. dropping out video or audio | 0 | 1 | 2 | 3 |  | 99 |
| Q3d. | Limitations on what we can cover in the visit, e.g. physical exam, showing printed materials or diagrams | 0 | 1 | 2 | 3 |  | 99 |
| Q3e. | Other (please specify) | 0 | 1 | 2 | 3 |  | 99 |

Q4. If more than one response selected as ‘1’, ‘2’, or ‘3’ in Q3] **What has been your main frustration with telehealth over the past few months? Please select one.**

[List Q3 items that = 1, 2, or 3]

D1. In what setting do you primarily work?

1 Private/Solo practice

2 Group practice

3 Hospital- or HMO-based practice

4 Other (please specify)

D2. Does your practice use an electronic health record?

1 No

2 Yes, we use an Epic electronic health record

3 Yes, we use a Cerner electronic health record

4 Yes, we use a different electronic health record (please specify)

5 Other (please specify)

D3. How old are you?

1 18-24 years

2 25-34 years

3 35-44 years

4 45-54 years

5 55-64 years

6 65-74 years

7 75+ years

D4. How long has it been since you graduated from medical school?

1 0-3 years

2 4-9 years

3 10-19 years

4 20-29 years

5 30 or more years

Q5. Would you like to share any other comments about telehealth and/or your experience using telehealth?

[Open-ended response field]
