# Supplementary material for: Telehealth Modality Preferences for Video and Voice-Only Visits Among US Clinicians and the Public: Cross-Sectional, Web-Based Survey Study
Source: J Med Internet Res. 2025 Jun 13;27:e72276. doi: 10.2196/72276 (PMC12180677; doi:10.2196/72276)
Supplement: Multimedia Appendix 2 [file jmir-v27-e72276-s002.docx]

**Appendix 2: Public questionnaire**

D8. What is the highest level of formal education you have completed?

1 Less than a high school degree

2 A high school diploma or GED

3 Technical training or certification

4 Some college

5 A college degree

6 A graduate or professional degree

D9. How would you describe the community where you currently live?

1 A large city

2 A suburb near a large city

3 A small city or town

4 A rural area

To avoid the spread of COVID-19, healthcare teams have increased their use of telehealth, where people talk to healthcare teams using the phone, smartphone, tablet or computer. We’d like to ask you a few questions about how to use telehealth in the future.

When we talk about ‘phone calls,’ we mean real-time sound-only connections on a landline or cell phone.

When we talk about ‘videoconferences,’ we mean real-time sound and visual connections on a computer, tablet, or smartphone.

| **Q1. Which platform would you prefer for each of the following scenarios:** [randomize item order] | | |
| --- | --- | --- |
|  | **Phone call** | **Videoconference** |
| 1. Attending my first appointment with a clinician who I don’t know | 1 | 2 |
| 1. Telling a clinician who I already know about a new problem | 1 | 2 |
| 1. Updating a clinician who I already know about a problem that we’ve talked about before | 1 | 2 |

**Q2. How important are each of these things for a telehealth experience?**

|  |  | **Not important** | **Somewhat important** | **Important** | **Extremely important** |
| --- | --- | --- | --- | --- | --- |
| **Q2a.** | Ease of using the platform (e.g. starting and ending the appointment, controlling the volume) | 0 | 1 | 2 | 3 |
| **Q2b.** | Flexibility in where I can take the appointment (e.g. at home, in the car, somewhere else) | 0 | 1 | 2 | 3 |
| **Q2c.** | Being able to hear my clinician clearly | 0 | 1 | 2 | 3 |
| **Q2d.** | Being able to see my clinician clearly | 0 | 1 | 2 | 3 |
| **Q2e.** | The clinician being able to hear me clearly | 0 | 1 | 2 | 3 |
| **Q2f.** | The clinician being able to see me clearly | 0 | 1 | 2 | 3 |
| **Q2g.** | Being able to see what’s on my clinician’s computer screen and review materials together | 0 | 1 | 2 | 3 |

**Q3. Have you had a telehealth appointment with a healthcare provider in the past year? Please select all that apply.**

1 Yes, by phone

2 Yes, by videoconference

3 No [sole allowed response]

Q4. [If Q3 !=3] **Have you had any of the following frustrations with telehealth?** (randomize item order a-d)

|  |  | No frustration | A little frustration | Some frustration | A lot of frustration |
| --- | --- | --- | --- | --- | --- |
| Q4a. | Downloading new technology | 0 | 1 | 2 | 3 |
| Q4b. | Figuring out how to use new technology | 0 | 1 | 2 | 3 |
| Q4c. | The technology not working properly, e.g. dropping out video or audio | 0 | 1 | 2 | 3 |
| Q4d. | Limitations on what we can cover in the visit, e.g. physical exam, reviewing printed materials or diagrams | 0 | 1 | 2 | 3 |
| Q4e. | Other (please specify) | 0 | 1 | 2 | 3 |

Q5. **What is your main frustration with telehealth? Please select one.**

[List Q4 items != 0]

D1. What gender do you identify with?

1 Male

2 Female

3 Non-binary

4 Prefer to self-describe: __________

5 Prefer not to say

D2. How often do you need to have someone help you when you read instructions, pamphlets, or other written material from your doctor or pharmacy?

1 Never

2 Rarely

3 Sometimes

4 Often

5 Always

D3. How old are you?

1 18-24 years

2 25-34 years

3 35-44 years

4 45-54 years

5 55-64 years

6 65-74 years

7 75+ years

8 Prefer not to say

D4. What is your current employment status? [ALLOW MULTIPLE RESPONSES]

1 Full time paid work (40 or more hours per week)

2 Part time paid work (less than 40 hours per week)

3 Unemployed and looking for work

4 Unemployed and not looking for work

5 Retired

6 Student

7 Other (please specify)

D5. Which of the following internet-connected devices do you have at home? Please select all that apply.

1 Smartphone

2 Personal computer (laptop or desktop)

3 Tablet computer

4 Other (please specify)

5 No internet-connected devices [sole response allowed]

D6. Which of the following services do you have at home? Please select all that apply.

1 Landline phone

2 Mobile/Cell phone

3 Mobile phone data (internet)

4 Dial-up internet (uses a phone line to connect)

5 Broadband internet (includes wifi and ethernet connections)

D7. Overall, how often do you use the internet?

1 Several times a day

2 About once a day

3 3-5 days a week

4 1-2 days a week

5 Every few weeks

6 Less often

7 I don’t have regular internet access

Q6. Would you like to share any other comments about telehealth and/or your experience using telehealth?

[Open-ended response field]
